# Change in the size of apical radiolucencies in adolescent’s mature maxillary incisors following retreatment with two regenerative endodontic techniques: a 12-month randomised clinical trial using volume-based cone-beam computed tomography

**DOI:** 10.1007/s00784-025-06344-y

**Published:** 2025-05-03

**Authors:** Norhan Khaled Omar Wahba, Sherif Shafik E. L. Bahnasy, Yassmin Mohamed ElMakawi, Paul M. H. Dummer, Venkateshbabu Nagendrababu, Giampiero Rossi-Fedele, Francesc Abella Sans, Damiano Pasqualini, Mario Alovisi, Mohammed Turky, Eman Farouk Ahmed, Ahmad Abdel Hamid Elheeny

**Affiliations:** 1 Demonstrator of Paediatric and Community Dentistry, Faculty of Oral and Dental Medicine, Nahda University, New Bani Suef, Egypt; 2 Lecturer of Oral Radiology, Faculty of Dentistry, The British University in Egypt, Al Shorouk City, Egypt; 3 Lecturer of Paediatric and Community Dentistry, Faculty of Oral and Dental Medicine, Nahda University, New Bani Suef, Egypt; 4 School of Dentistry, College of Biomedical and Life Sciences, Cardiff University, Cardiff, UK; 5 Department of Preventive and Restorative Dentistry, College of Dental Medicine, University of Sharjah, Sharjah, UAE; 6 Adelaide Dental School, University of Adelaide, Adelaide, South Australia Australia; 7 Department of Endodontics, School of Dentistry, Universitat International de Catalunya, Sant Cugat del Valles, Barcelona, Spain; 8 Department of Surgical Sciences, Dental School, University of Turin, Turin, Italy; 9 Department of Endodontics, Faculty of Dentistry, Minia University, Minia, Egypt; 10 Department of Endodontics, Faculty of Dentistry, Sphinx University, Assiut, Egypt; 11 Microbiology and Immunology Department, Faculty of Pharmacy, Sohag University, Sohag, 82524, Province Egypt; 12 Paediatric and Community Dentistry, Faculty of Dentistry, Minia University, Province, 61519 Minya Egypt; 13 Paediatric and Community Dentistry, Faculty of Dentistry, Sphinx University, Asyut Al Gadida City, Egypt

**Keywords:** Cone-Beam Computed Tomography, Regenerative Endodontics, Platelet-Rich Fibrin

## Abstract

**Objectives:**

The primary aim of this randomised clinical trial was to compare the one year clinical and radiographic outcome of mature permanent central incisors with periapical radiolucencies in adolescents after root canal retreatment using two regenerative endodontic procedures (REPs) with revitalization using induced blood clot formation (BC) or platelet-rich fibrin (PRF) evaluated with cone-beam computed tomography (CBCT). The secondary aim was to assess the responses of the teeth to thermal and electric pulp tests.

**Materials and methods:**

Fifty-four root filled maxillary central incisors with post-treatment endodontic disease and periapical radiolucencies in 48 adolescents were allocated into two groups (*n* = 27) using permuted block randomisation. The teeth in one group were root canal retreated with induced BC formation and teeth in the other with PRF. At baseline and at one year, teeth were evaluated clinically and radiographically using periapical radiographs and CBCT scans. Changes in the maximum diameter and volume of the periapical lesions were assessed and pulp sensibility was assessed at one year using thermal and electrical tests. Differences in lesion diameter and volume between the two groups were tested using the Mann–Whitney U test. A linear regression model explored the relationship between independent variables and lesion size. The significant level was set at 5%.

**Results:**

Reduction in periapical lesion size in the BC and PRF techniques occurred in 85% and 100% of teeth, respectively, with no significant difference. In the BC group, the mean lesion volume diminished from 0.33 ± 0.18 cm^3^ to 0.13 ± 0.20 cm^3^, while the mean volume of lesions in the PRF group decreased from 0.27 ± 0.16 cm^3^ to 0.04 ± 0.06 cm^3^ with no significant difference between the groups (*P* > 0.05). Significantly more teeth responded positively to thermal (*P* = 0.028) and electric (*P* = 0.032) tests in the PRF group compared to the BC group.

**Conclusions:**

REPs using BC or PRF techniques when retreating root canal-treated mature permanent central incisors in adolescents with apical radiolucencies had comparable clinical and radiographic outcomes one year following treatment associated with significantly more positive responses to thermal and electric pulp tests in the PRF group.

**Clinical relevance:**

Retreatment of mature permanent teeth with apical periodontitis using regenerative endodontic procedures (REPs) is a new and promising approach. REPs with platelet-rich fibrin (PRF) and revascularization techniques provided high and comparable clinical and radiographic success rates.

## Introduction

Root canal retreatment or endodontic surgery are the preferred management options for teeth with post-treatment endodontic disease [1]. Despite the favourable outcomes of root canal retreatment, regenerative endodontic procedures (REPs) have been reported to restore pulp-like tissues and initiate the healing of periapical lesions [2]. REPs have the added advantage of the development of regenerated vital neurovascular pulp-like tissues [3] and potentially allow the maintenance of a range of repair mechanisms, immunological response and proprioceptive functions [4].

The predictable outcomes of REPs in the treatment of immature teeth with necrotic pulps has led to its application in the treatment of mature permanent teeth with irreversible pulpitis, pulp necrosis, and periapical lesions [2, 5–7]. REPs are a guided tissue biological process that prompts the repair of damaged tooth tissues [7–9]. However, the concept of REPs depends on the integration of the three basic constituents of tissue engineering: mesenchymal stem cells (MSCs), scaffolds, and growth factors [10].

Stimulating blood from the periapical tissues to enter disinfected root canals results in blood clot (BC) formation. The BC scaffold provides a homing stimulus for undifferentiated MSCs and is rich in growth factors that prompt cellular proliferation and differentiation [11]. However, the BC technique does not always result in sufficient bleeding from what are often severely compromised apical tissues, which results in low levels of the essential growth factors and cytokines [12, 13]. The width of the apical foramen and dimensions of the root canal required for successful REPs is controversial [14], as a result, one of the concerns for the use of REPs in permanent mature teeth is related to the often small apical diameters of the canals and foramina [15]. Another problem is the type of cells that migrate into the canal, which may be non-stem cells from the apical papilla (non-SCAP) that results in the proliferation and differentiation and deposition of hard tissues in the root canal rather than pulp tissue [16].

In an attempt to overcome the potential drawbacks of the BC method, alternative scaffold systems have been introduced with the aim of promoting the abundant controlled release of growth factors. Platelet-rich fibrin (PRF) is an autologous scaffold system representing the second generation of platelet concentrate. In contrast to BC, which is formed mainly of approximately 95 volume% RBCs, 5 volume% platelets, and < 1 volume% WBCs, PRF provides a 210-fold increase in the platelet and fibrin concentration compared to the whole blood input volume (55 volume% plasma and 45 volume% formed elements ‘platelets, leucocytes, and erythrocytes) [17]. PRF scaffolds have the ability to form a solid complex fibrin network that protects the platelet scaffold from degradation and entraps proinflammatory mediators and cytokines. This supports the continuous release of the many cytokines required with higher viability for several weeks [18–20].

Cone-beam computed tomography (CBCT) permits accurate diagnosis of periapical lesions with two-times higher sensitivity compared to traditional radiographic imaging approaches [21] and addresses the limitations of periapical radiographs (PAs) by overcoming the problem of superimposed structures and detecting small lesions that are often missed by PAs. A systematic review concluded that the accuracy of periapical lesions diagnosis was significantly superior when using CBCT compared to PAs [22]. Indeed, it has been reported that CBCT images are able to identify approximately one-third more periapical lesions than PAs, in addition to detecting lesion volumes ranging from 6.7 mm^3^ to 41.3 mm^3^, which cannot be identified in PAs [23]. Segmentation tools to monitor the healing of periapical lesions have become established, providing valuable data regarding volumetric measurements [24]. The segmentation of CBCT scans has confirmed its accuracy and reliability using ex vivo models [25, 26].

Limited data is available on the use of REPs during root canal retreatment of mature teeth. Root canal retreatment of mature teeth using REPs was assessed in a randomized controlled trial of adolescents aged 11 to 17 years (mean age of 15.29 ± 1.64 years) [27] when the treatment of 33 permanent incisors with periapical pathosis using REPs resulted in a 93.9% success rate at one year. The frequencies of positive EPT readings were 55% after 12 months of follow-up.

The primary aim of the present trial was to compare radiographic changes in periapical lesion size of mature permanent maxillary central incisors in adolescents following two regenerative endodontic techniques at one year. A secondary aim was to assess the responses of retreated teeth to thermal and electric pulp tests. The null hypothesis proposed no difference in the reduction of lesion size between revitalization (BC) and the PRF scaffold techniques, clinical signs and symptoms, and positive pulp responses of root canal retreated permanent central incisors with mature apices.

## Materials and methods

### Ethical approval

**Fig. 1:**
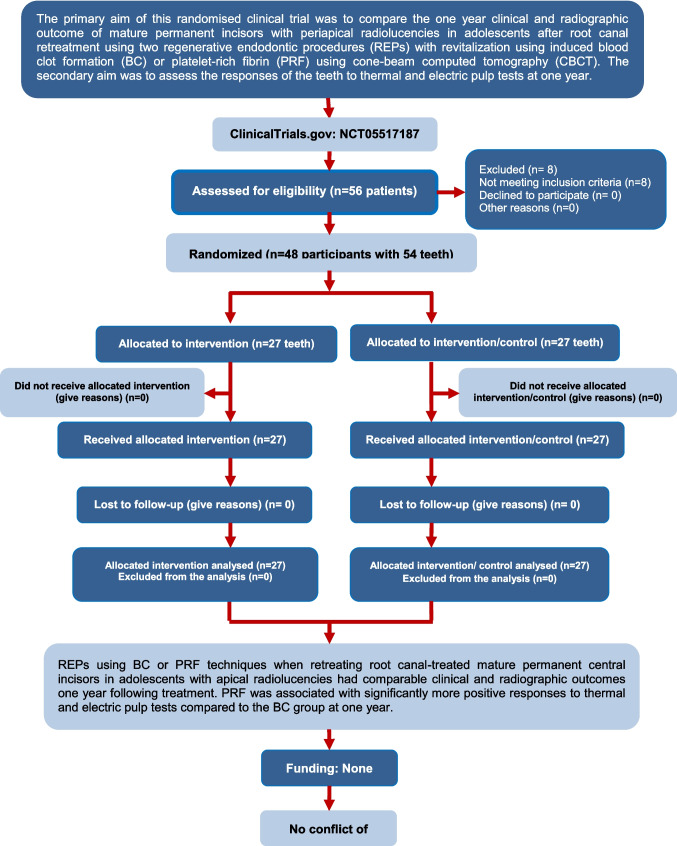
CONSORT 2010 flowchart of the trial

The manuscript was written in compliance with the guidelines of CONSORT 2010 statement (Fig. 1). The trial was approved by the ethics committee of the local institution and registered at clinicaltrial.gov on August 31, 2022. The trial started on August 31, 2022, and finished on July 29, 2024. Before inclusion in the study, the parent or legal guardian of each participant signed an informed consent after a detailed explanation of the study's purpose, procedures, benefits, potential side effects, and alternative treatment approaches in case of failure.

### Sample size

An effect size of 0.38 was calculated according to the findings of a pilot study that included twenty teeth (10 per group) from 16 patients. The periapical lesion volume (PALV) means of PRF and BC were 0.26 cm^3^ and 0.41 cm^3^, respectively, with a pooled standard deviation of 0.39. At an alpha level of significance of 5 percent and a power of 80 percent, a repeated measure ANOVA was used to calculate the sample size. Considering the cluster effect obtained from the inclusion of more than one tooth in the same participant, the sample was increased by an additional 20% with a further 10% added to account for potential drop-outs. Therefore, a total of 54 teeth were deemed sufficient.

### Participant selection

Healthy adolescents of both genders aged 11 to < 18 years old and categorized as class I ASA (American Society of Anesthesiologists) with no history of allergies, or systemic, or genetic conditions were enrolled. Root canal-treated maxillary central incisors with single straight roots and canals, and fully developed apices with apical foramen diameters ranged from 0.2 mm $-$ 0.4 mm and root lengths of 11 $-$ 13 mm were included. All the selected teeth were asymptomatic apical periodontitis. As post-treatment endodontic disease can occur at various time points following the initial RCT, and the underlying reasons for failure may differ between short- and long-term cases, the primary root canal treatments for the selected cases were performed at least 1 $-$ 2 years prior to conducting the trial. The potential causes of post-endodontic disease were investigated to rule out evidence of catastrophic fractures in the crown and/or the root and determine the possibility of retreatment and the best management options. The analysis revealed that the most common causes of the post-treatment disease included the absence of a permanent restoration, defective integrity of the restoration (such as fractured restoration or fracture of the tooth tissue adjacent to the restoration), and inadequate root canal filling. Unrestorable teeth and those with a history of trauma, fractured roots, need for post and core placement or periodontal involvement, grade III mobility, irretrievable fractured instruments, calcified root canals, or pulp stones were excluded. Furthermore, cases with failure to remove all the existing root canal filling material or extrusion of material beyond the canal terminus were excluded.

### Randomization, allocation, and blinding

To ensure comparable groups, preoperative lesion sizes were classified according to the cone-beam computed tomographic periapical volume index (CBCTPAVI) [21]. A permuted block randomization was carried out using computer-generated software (https://www.sealedenvelope.com). An independent researcher who was concealed from the purpose and procedures of the trial generated the randomization sequence. An independent assistant who was blind to the treatment protocols, codes, and randomization sequence wrapped double-folded sheets containing the treatment codes in identical, sequentially numbered opaque sealed envelopes. For each block, four envelopes (two envelopes per group, either PRF or BC that was matched according to their lesion sizes) were shuffled and placed into four identical containers. One envelope was chosen at random by the participant's guardian at the time of the procedures. Independently, the radiographic and clinical assessments of all cases were carried out by two calibrated specialists who were masked to the treatment groups; it was not possible to blind the patient or the operator.

### Clinical procedures

For both groups, the clinical steps of both treatments were performed over two visits according to the REPs recommended by the American Association of Endodontists (AAE) [28]. A rubber dam was applied after the tooth had been anaesthetised with articaine hydrochloride 4% and epinephrine 1:100,000 (Septocaine®, SEPTODONT Ltd. Paris, France). Subsequently, the entire coronal restoration was removed, and the access cavity was adjusted according to the standard guidelines of access cavity preparation to achieve straight-line access to the canal orifice [29]. The existing root canal filling material was removed using an appropriate-sized stainless-steel H-file (MANI Inc., Tochigi, Japan) until no residue of material were present along the shaft of the file (no chemical solvents used) [30]. A PA was exposed to ensure that there were no remnants of root canal filling. To determine the working length, an electronic apex locator (DentaPort ZX, J. Morita Corp., Kyoto, Japan) with the assistance of PA was used according to the guidelines of the European Society of Endodontology [31]. The root canal was disinfected using alternate irrigation with 20 mL of 1.5% NaOCl for 5 min and 20 mL of 17% ethylenediaminetetraacetic acid (17% EDTA) (Prevest, DenPro, Jammu, India) for 5 min, and then the root canal was flushed with 5 mL of 0.9% saline. A 30-gauge side-vented needle (Zhucheng Binfei Medi-Tek Co., Ltd. Shandong, China) was inserted 1 $-$ 2 mm short of the working length. The canal was dried with suitable-sized paper points (Meta Biomed, Korea), and a non-settable calcium hydroxide (UltraCal, Ultradent, Utah, USA) intracanal medication was injected into the root canal space. The access cavity was restored with a light-cured resin glass ionomer (GC Fuji II LC, Tokyo, Japan). Patients were scheduled for a second appointment three weeks later. At the second appointment, the tooth was isolated after being anaesthetized using labial infiltration with 3% mepivacaine plain local anaesthesia (without a vasoconstrictor) (Scandonest®, Septodont, Saint-Maur-des-Fosses, France). The root canal was irrigated with 5 mL of 0.9% saline and 20 mL of 17% EDTA. The EDTA was passively activated for successive 3 cycles of 20 s each using an ultrasonic, non-cutting tip (E1 Irrisonic tip; Helse Dental Technology, São Paulo, SP, Brazil).

#### BC group

The apical foramen was prepared to size 35, than a sterile stainless-steel size 35 K-file was extended 2 mm beyond the apical foramen to provoke bleeding from the periapical region in order to fill the root canal space up to 3 $-$ 4 mm below the cementoenamel junction (CEJ). If the induction of a significant amount of bleeding into the root canal space couldn't be achieved, the tip of the manual endodontic file was slightly bent after being dipped into 17% EDTA.

#### PRF group

**Fig. 2:**
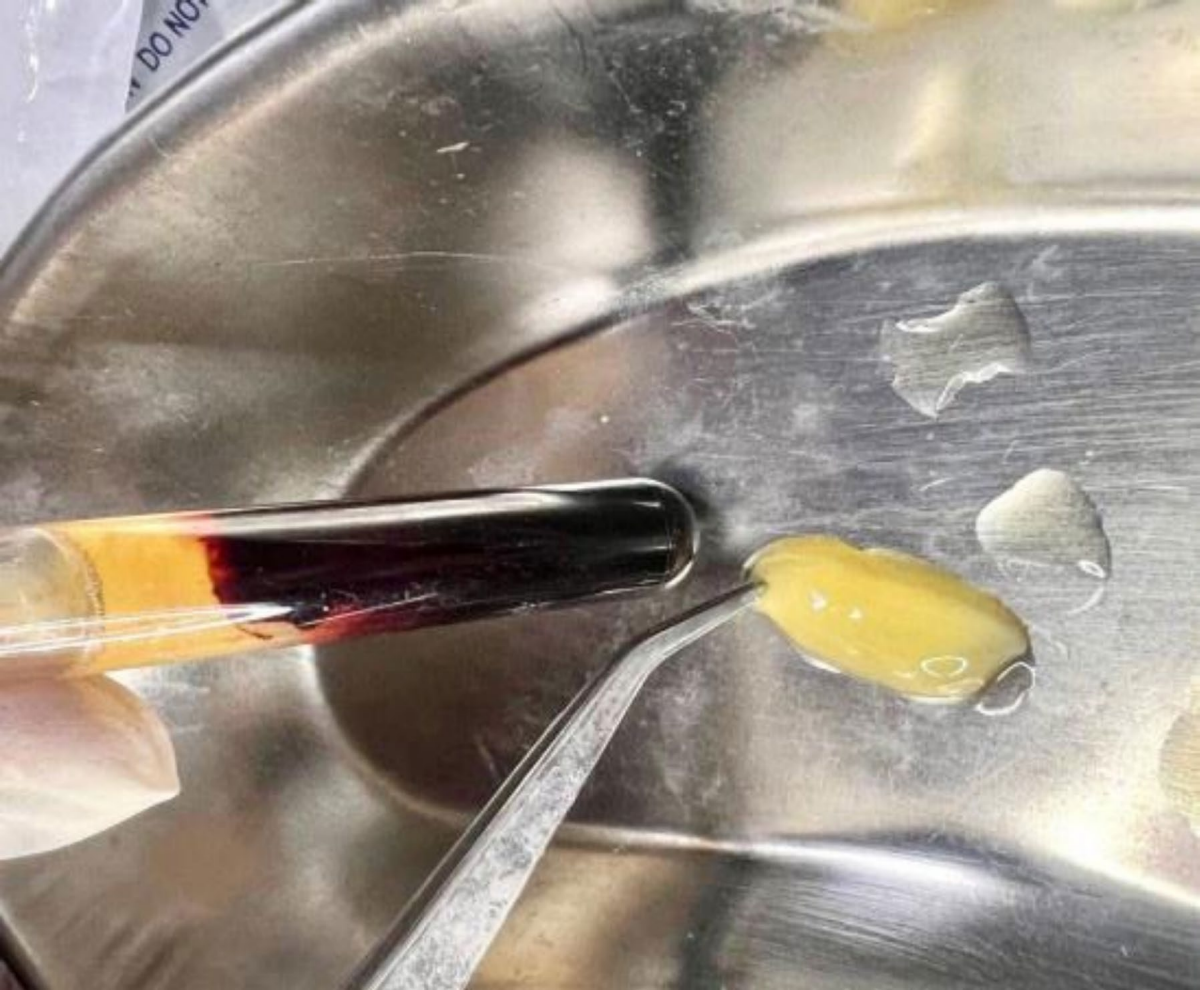
Isolated PRF scaffold

A 5 mL blood sample using a sterile hypodermic needle (Luer-Slip type, El Dawlia ico nos. 19 and 67) was collected through venepuncture of the antecubital vein. With no addition of anticoagulants, the venous blood sample was drawn into sterile, plain-type single-use centrifuge tubes (JIANGSU HXRT MD Co., Ltd. Taizhou, China). The tubes were centrifuged (MSLZL09 MedGroup Guangzhou Medsinglong Medical Equipment, GuangZhou, China) for 10 min at 3000 revolutions per minute (rpm). The blood component was separated into three distinctive layers: acellular platelet-poor plasma at the top, PRF layer at the middle, and a red blood cell layer at the bottom. Using an HTS 171c66.25 curved stainless steel college tweezer, the middle fibrin-gelatinous yellow fraction was separated from the lowest red blood cell layer (Fig. 2). The isolated PRF clot was dragged over sterile gauze to remove the surplus. Similar to the BC approach, intracanal bleeding was induced after preparing the apical foramen was to size 35 but without allowing a blood clot to form. The PRF clot fragments were placed immediately into the root canal using a small hand plugger (Dentsply Maillefer, Ballaigues, Switzerland) at working length and up to 3 mm below the CEJ [7].

### Tooth restoration

For both groups, a 3 mm layer of white MTA (Vericom Well root PT., Seoul, South Korea) was placed against a collagen-resorbable matrix (Collacone®, Botiss Biomaterials, Berlin, Germany) over either the BC or PRF clot. Then, a moist cotton pellet was placed over MTA and the access cavity was restored temporarily with a reinforced glass ionomer material (GC Fuji II LC, Tokyo, Japan). On the next day, the cotton pellet was removed, and a layer of reinforced glass ionomer was applied as a base over the MTA, followed by a resin composite (3M ESPE, Filtek Supreme Z350XT, Minnesota, USA) to restore the cavity.

### Radiographic assessment and analysis

#### Periapical radiographs (PAs)

Routine conventional periapical radiographs (PAs) were taken preoperatively and at 6 and 12 months to monitor the healing process. Intraoral periapical images were obtained using the Vista Scan system and software (DÜRR DENTALTM, Bietigheim-Bissingen, Germany) and the BelmontTM 2 X-ray machine with a focal spot size of 0.4 mm. For all patients, the radiographic parameters were set at 60 kVp, 6 mA, and 0.05 s. The parallel intraoral technique was used in which a silicone bite was used to standardize the film positions and the film holder was attached to a digital radiography phosphor plate, size 2 (3 $\times$ 4 cm).

#### CBCT images

CBCT scans were taken preoperatively and at 12 months. Based on the principle of"as low as reasonably achievable"(ALARA), the effective radiation dose of each CBCT scan was minimised by using a high resolution and small field of view (FOV) device [32]. The CBCT images were obtained using the Planmeca® Viso G7 CBCT machine (Planmeca Oy Asentajankatu 6 FIN- 00880 Helsinki, Finland), using same appropriate field of view for all patients 17 × 17 cm, voxel size 200 µm and (100 kV, 7.1 mAs) using ULD ultra-low dose acquisition protocol. The ULD ultra-low-dose 3D image acquisition protocol and all radiation protection measures at baseline and after 12 months of follow-up were considered. All images were interpreted with Planmeca Romexis® software (Version 6.3. Planmeca Oy Asentajankatu 6 FIN- 00880 Helsinki, Finland). Images were viewed using a Dell monitor (22’’ Full HD 1920 × 1080 display) in a dimly lit room. On the multiplanar (MPR) screen, coronal, axial, and sagittal views were reoriented to make the periapical area of measured teeth in the centre of the image. In the axial view and cross-section view, the maximum diameter of the lesion was measured by a distance measurement tool to an accuracy of 0.01 mm. To assess the change in periapical lesion size, each lesion volume was segmented at baseline and the end of the study. For segmentation, the lesion was viewed in one plane. The image threshold was adjusted to provide a suitable representation of the bone. At this point, plotting out the border of the lesion slice by slice has initiated. Once all slices had been completed, the measurements were interpolated, and the volume was calculated. This was performed by expert in oral and maxillofacial radiology with a PhD in oral and maxillofacial radiology in collaboration with a PhD endodontic expert.

#### Pulp sensibility

Thermal and electric pulp tests were performed at 6 and 12 months following treatment. Tooth isolation was achieved by placing cotton rolls in the muccolabial fold, and then teeth were dried with sterile gauze. For comparison, healthy adjacent teeth were tested first. Thermal pulp testing (TPT) was conducted by applying a small cotton pellet sprayed with Green Endo-Ice refrigerant (Coltene/Whalkedent Inc., Cuyahoga Falls, Ohio, USA) for five seconds. The readings of the electric pulp testing (EPT) (Parkell D640 Digitest II Pulp Vitality Tester, Brentwood, New York, USA) were recorded by applying the probe tip of the EPT, which was covered by a thin layer of toothpaste. The middle third of the labial surface was tested with the refrigerant-saturated cotton pellet and the electric probe. Two dentists checked the pulp response at each follow-up interval independently. The degree of inter-examiner agreement using Cohen's kappa coefficient (κ) was 1.00.

### Definition of outcomes

At the end of follow-up period, each retreated tooth was assessed for a reduction in the diameter of the periapical lesions (PALD) and periapical lesion volume (PALV) using the CBCT scans. In addition, the absence of inflammatory root resorption, the absence of clinical signs and symptoms (pain, tenderness to percussion, swellings, or sinus tract) was also assessed.

#### Standardization and calibration measures

The treatments were performed by a single operator with eight years of clinical experience in REPs. Two independent experts assessed the periapical radiographs and CBCT scans, and Cohen's kappa coefficient (κ) was calculated to determine the degree of inter-examiner agreement and values calculated for both PAs and CBCTs (κ $=$ 0.90). The inter-examiner agreement of the segmentation of lesion volume at baseline and after 12 months was classed as excellent (κ = 0.92).

### Statistical analysis

Data were analysed using the chi-square test and the Fisher exact test. Quantitative statistics were tested for the normality assumption and variance homogeneity using the Kolmogorov–Smirnov and Shapiro–Wilk tests. The mean age of the participants was compared using an independent sample *t-test*. Since, the PALD and PALV scores were not normally distributed, the non-parametric Mann–Whitney U test was considered to compare the PALD and PALV medians of the two REP techniques at baseline and the PALD and PALV medians of the lesions at 12 months.

Considering the means of PALD and PALV as cut-off points for lesion diameters and lesion volumes, the PALD and PALV values were dicotomised at baseline. A linear regression model was used to explore the relationship between independent variables and lesion size at the end of follow-up (i.e., PALV). A generalised linear model (GLM) was used to calculate the effect of time and the regenerative endodontic technique on PALD and PALV after considering the cluster effect obtained from more than one tooth from the same participant. The alpha significant level was set at 5% (*P* ≤ 0.05) and a 95% confidence interval (*CI*).

## Results

**Fig. 3:**
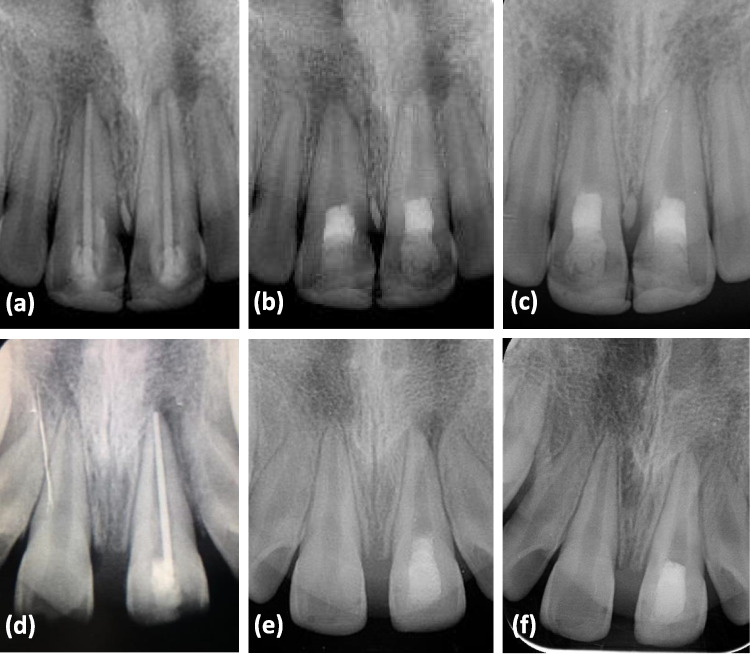
Periapical radiographs of two patients retreated with platelet-rich fibrin (PRF) (**a**, **b**, and **c**) and revascularization with induced blood clot formation (BC) (**d**, **e**, and **f**). **a** Preoperative radiograph of a 14-year-old male with periapical radiolucency related to root canal retreatment on maxillary central incisors; **b** and **c** continuous reduction in size of periapical radiolucency after 6 months and 12 months of follow-up; **a** preoperative radiograph of a 13-year-old female with periapical radiolucency related to root canal retreatment on a right maxillary central incisor; **b** and **c** continuous reduction in size of periapical radiolucency after 6 months and 12 months of follow-up

Fifty-four maxillary central incisors in 48 adolescents were included after the examination of 67 teeth. No significant differences in the demographic characteristics of the participants between groups were found (Table 1). Both groups had comparable clinical and radiographic outcomes, with no significant difference. The PAs revealed a reduction of lesion size in the PRF and BC techniques of 100% and 85% respectively (Fig. 3). In the BC group, after 6 months, three teeth (11%) were associated with swelling and sinus tract formation, and an increase in the size of the periapical radiolucency. An additional tooth was clinically within normal limits but radiographically had an increased PALD. These teeth were subsequently referred for root canal retreatment.

**Table 1:** Demographic features and radiographic and clinical outcomes of the two regenerative techniques, blood clot (BC) and platelet-rich fibrin (PRF)

Variables	BC	PRF	*P*-value
Gender			
Males	18(67)	19(70)	0.77
Females	9(33)	8(30)	
Age (years)			
Mean ± SD	14.74 ± 1.35	14.44 ± 1.19	0.40
Tooth type			
Maxillary right central incisor	17(63)	13(48)	0.21
Maxillary left central incisor	10(37)	14(52)	
PALD (mm) means of lesions			
Baseline	4.01 ± 1.71	3.70 ± 1.89	0.31
12 months	1.64 ± 2.13	.89 ± 1.30	0.75
PALV (mm^3^) means of lesions			
Baseline	333 ± 180	270 ± 160	0.06
12 months	130 ± 200	400 ± 600	0.09
Radiographic outcomes			
Healing in progress	23(85)	27(100)	0.24
Diseased	4(14)	0(0)	
Clinical outcomes			
Success	24(88)	27(100)	0.24
Failure	3(11)	0(0)	

Chi-square test for gender and tooth type; Fisher exact test for clinical and radiographic success rates; Independent t-test for age. *P*-value set at ≤ 0.05

**Fig. 4:**
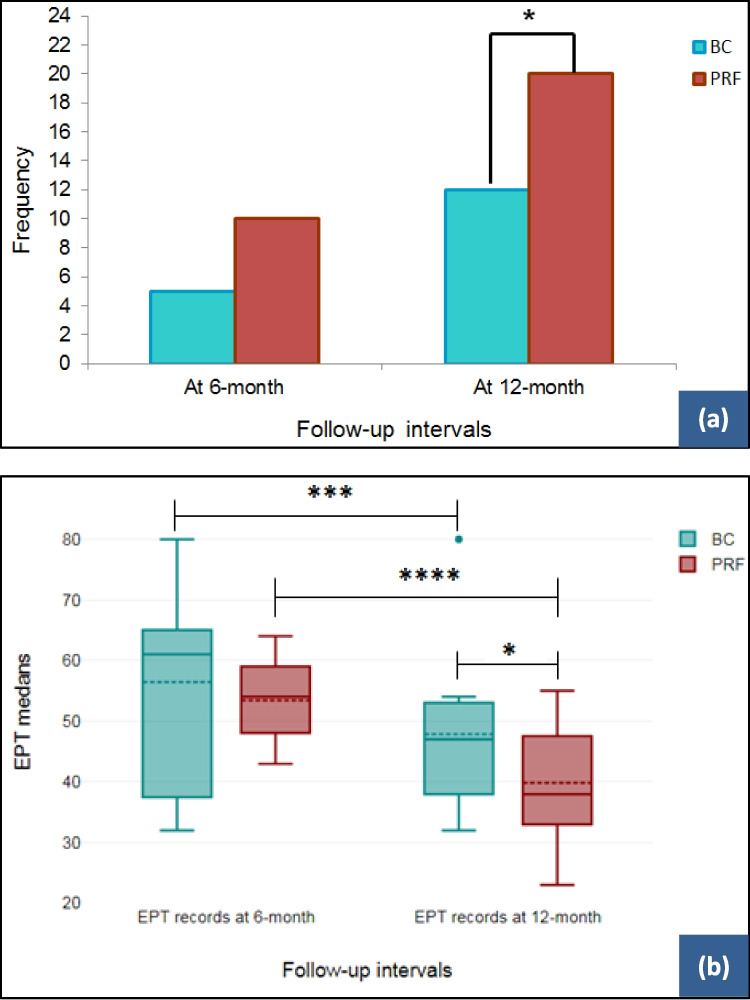
Response of retreated teeth to (**a**) thermal pulp testing (TPT) and (**b**) electric pulp testing (EPT) after 6 and 12 months after regenerative endodontic procedures (REPs). **a** Distribution of positive TPT responses of platelet-rich fibrin (PRF) and revascularization with induced blood clot (BC) retreated teeth. The difference between the two REP techniques is statistically significant after 12 months of follow-up (Fisher exact test). **b** Median EPT scores after 6 and 12 months. The difference between the two REPs is statistically significant after 12 months of follow-up (*Mann–Whitney U* test). Intragroup comparison showed a significant decrease in the median EPT scores in both REP techniques (Wilcoxon sign-rank test). *P ≤ 0.05, ** *P* ≤ 0.01, ***P ≤ 0.001, **** *P* ≤ 0.0001

Significantly more teeth in the PRF group responded positively to TPT after one year of follow-up (*P* = 0.028) (Fig. 4a). The number of teeth responding to EPT testing at 12 months was significantly higher in the PRF group (*P* = 0.032). The mean EPT reading in the PRF group was 39.89 ± 9.07 (median = 38) compared to the BC group with a mean of 47.81 ± 13.67 (median = 47) (Fig. 4b).

The independent variables (gender, age, tooth type, and tooth position) had no significant effect on the clinical outcome of the regenerative endodontic techniques. Likewise, the preoperative lesion diameter and lesion volume had no significant impact on the outcome of the REPs (Table 2).

**Table 2:** The effect of variables on the clinical success of the two regenerative techniques at 12 months

Variables	Success	Failure	*P*-value
Gender			
Males	35(95)	2(5)	1.00
Females	16(94)	1(6)	
Age (years)			
Mean ± SD	13.69 ± 1.22	14.33 ± 1.15	0.12
Tooth type			
Maxillary right central incisor	28(93)	2(7)	1.00
Maxillary left central incisor	23(96)	1(4)	
PALD (mm) of lesions undergoing healing			
Mean ± SD	3.85 ± 1.77	3.93 ± 1.92	0.94
PALV (mm^3^) of lesions undergoing healing			
Mean ± SD	290 ± 170	400 ± 180	0.32

Fisher exact test for gender and tooth type; Independent t-test for age, PALD, and PALV. *P*-value set at ≤ 0.05

The CBCT scans revealed that the PALD and PALV at baseline and the decrease of lesion dimensions and volumes after one year of follow-up in the BC and PRF groups were not significantly different.

**Fig. 5:**
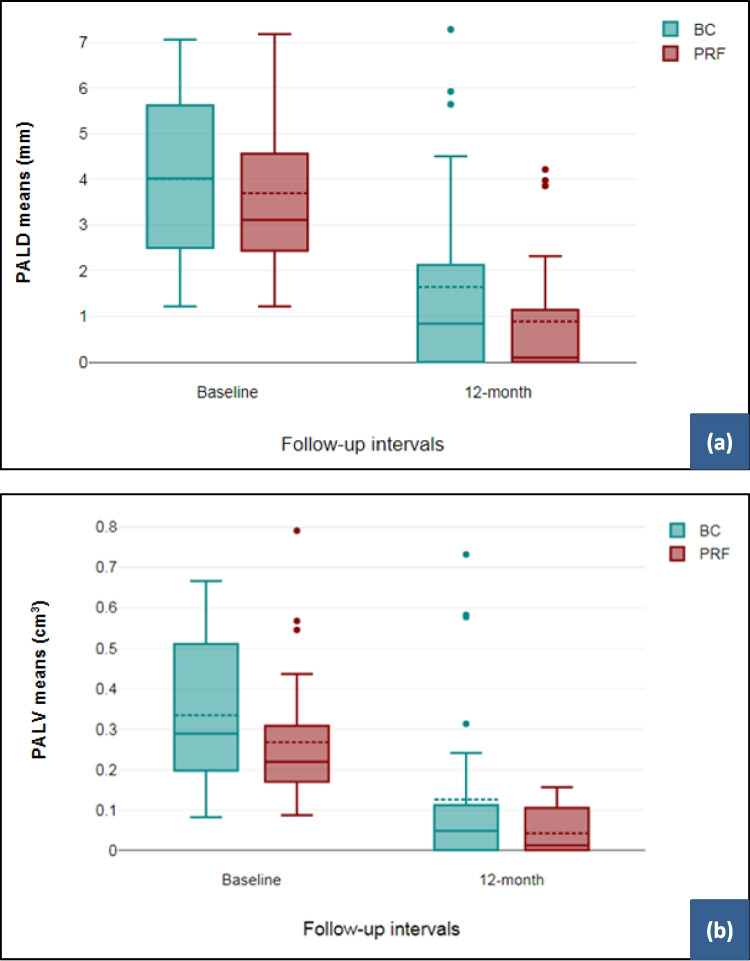
Cone-beam computed tomography (CBCT) median scores of **a** periapical lesion diameters (PALD). **b** Periapical lesion volumes (PALV). There was no statistically significant difference between the two REP techniques (*Mann–Whitney U* test)

**Fig. 6:**
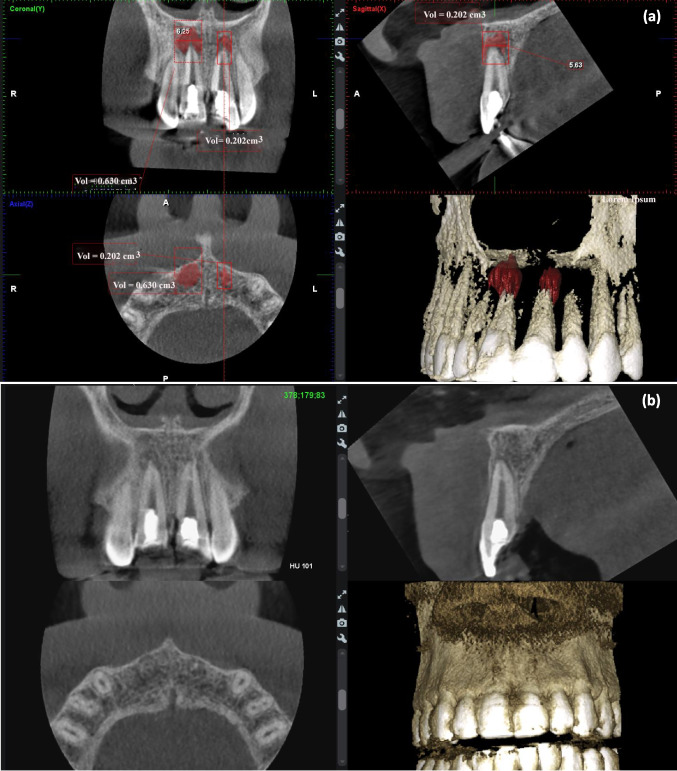
Cone-beam computed tomography (CBCT) scans of periapical lesion diameters (PALD) and periapical lesion volumes (PALV) of maxillary left and right central incisor teeth, respectively of a 14-year-old male retreated with platelet-rich fibrin (PRF) at **a** baseline; the maxillary left central incisor with PALD = 5.63 mm and PALV = 0.202 cm^3^, and the maxillary left central incisor with PALD = 5.63 mm and PALV = 0.202 cm^3^. **b** PALD = 0.00 mm and PALV = 0.00 cm^3^ after 12 months of follow-up

**Fig. 7:**
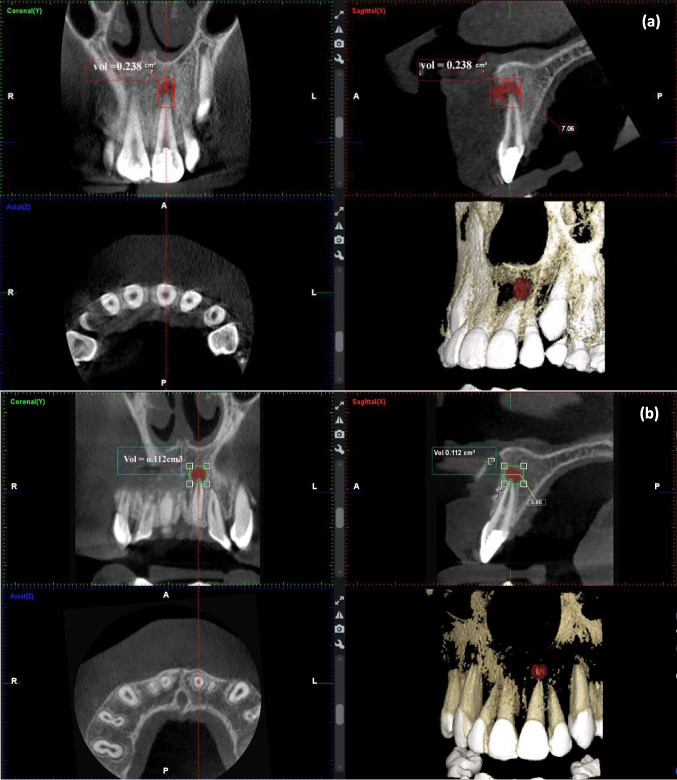
Cone-beam computed tomography (CBCT) scans of periapical lesion diameters (PALD) and periapical lesion volumes (PALV) of the maxillary left central incisor of a 13-year-old female retreated with induced blood clot (BC) at **a** baseline; the maxillary left central incisor with PALD = 7.06 mm and PALV = 0.238 cm^3^. **b** Reduction of periapical lesion size with PALD = 3.85 mm and PALV = 0.112 cm^3^ after 12 months of follow-up

Data in Fig. 5 shows the absolute mean values of PALD and PALV with no significant difference between the two REP techniques at the baseline and after 12 months. The change in the PALD means at the baseline and after 12 months in the BC and PRF groups were 2.37 ± 1.26 mm and 2.81 ± 1.29 mm, respectively (*P* = 0.21). The change in the PALV means at the baseline and after 12 months in the BC and PRF groups were 0.20 ± 0.09 cm^3^ and 0.23 ± 0.15 cm^3^, respectively (*P* = 0.38). An example of CBCT of two cases shows dimension in the PALD (Fig. 5) and PALV (Figs. 6 and Fig. 7) after retreatment with BC and PRF techniques.

According to the GLM, the reduction in lesion diameter and volume at the end of follow-up was 1.11 times (95% *CI* of 1.04; 1.20) and 1.18 times (95% *CI* of 1.03; 1.34) compared to the preoperative PALD > 3.85 mm and PALV > 0.32 cm^3^, respectively (Table 3). The effectiveness of the REPs over the follow-up period (i.e., time) had a significant effect on both PALD and PALV. On the other hand, there was no significant difference in the change of PALD and PALV between the two regenerative endodontic treatments over time (Table 4).

**Table 3:** The effect of variables on the periapical lesion volume (PALV) of lesions undergoing healing at 12 months using a linear regression model

Independent variables	Estimate	95% CI	*P*-value
Gender			
Male	0.98	0.90; 1.06	0.57
Female	1		
Age (years)			
≤ 15.50	1.06	0.98; 1.14	0.52
> 15.50	1		
Tooth type			
Maxillary right central incisor	1.10	0.96; 1.26	0.18
Maxillary left central incisor	1.18	0.99; 1.16	0.07
PALD at baseline (mm)			
> 3.85	1.11	1.04; 1.20	**0.004**
≤ 3.85	1		
PALV at baseline (cm^3^)			
> 0.32	1.18	1.03; 1.34	**0.011**
≤ 0.32	1

**Table 4:** The effect of time and technique (groups) on the periapical lesion diameter (PALD) and periapical lesion volume (PALV) of lesions undergoing healing at 12 months using a generalised linear model (GLM)

Effect size (ES)	PALD of lesions undergoing healing	PALV of lesions undergoing healing
Time	0.993	0.999
*P*-value	**0.050**	**0.020**
Technique	0.856	0.992
*P*-value	0.246	0.060

## Discussion

The treatment of permanent with necrotic pulps and closed apices using REPs has recently become a focus of attention. However, tracking the healing of periapical lesions of mature teeth with REPs using volume-based CBCT has not been reported. Therefore, the aim of the present trial was to compare changes in periapical lesion size after retreatment of mature permanent maxillary incisors in adolescents with two REPs techniques (i.e., induced BC versus PRF) as well as tracking the clinical and radiographic healing outcomes. The secondary aim was to assess the responses of the teeth to thermal and electric pulp tests at one year. Clinical outcomes and the reduction the lesion volume in both REPs groups was comparable with significantly more positive responses to thermal and electric pulp tests in the PRF group compared to the BC group.

For standardization and to rule out potential confounders, the design of the study was given considerable thought. For example, only a narrow age range was included to minimise the physiological changes and anatomical variations in terms of the root length and apical foramen position and width. Indeed, it is known that increasing age has the negative impact on the regenerative potential of MSCs. The regenerative potential of PDLSCs extracted from aged dogs demonstrated declining proliferative and trafficking abilities compared to those of young dogs [33]. Likewise, the proliferative and migration capacities of PDLSCs in humans have been reported to be influenced by age [34, 35]. Furthermore, aging could affect the position of the apical foramen, which might influence migration of MSCs from the apical region [6] and lead to variations in the pulp response to EPT and TPT. Additionally, in the present study, the participant's age range was likely to provide comparable levels of stem cells in the BC [36]. On the other hand, Arslan et al. [5] reported that age had no significant impact on the healing of periapical lesions. However, as a result of the lack of sufficient evidence of the aging effect on the efficacy of regenerative power, more studies are necessary to clarify this relationship.

Another measure of relevance was retreating only mature maxillary central incisors with single roots and root canals. The retreated teeth included were matched in terms of the preoperative lesion size and comparable apical foramen diameters in the PRF and BC groups and only teeth with small apical foramen diameters were included. According to EL-kateb et al., apical foramen diameters of 0.3 mm were sufficient for migration of stem cells to achieve successful revascularization in primary root canal treatment of mature maxillary incisors [6]. The effect of apical foramen diameter on the success of regenerative endodontic procedures has been reported by Fang et al. [37] who postulated a success rate of 90% of mature permanent teeth with apical foramen diameters less than 0.5 mm. However, there is no evidence regarding the recommended apical foramen diameter required to achieve successful REPs.

To track the progression of periapical lesions and monitor periapical pathosis in adolescents, the American Academy of Pediatric Dentistry (AAPD) considers CBCT as a useful adjunct diagnostic tool [38]. In the current study, preoperative CBCT scans were performed not only to determine the actual size of the periapical lesions but also to exclude the possibility of vertical root fractures, overextended root canal filling material, fractured instruments, ledge formation and root perforations [39]. There are no guidelines regarding the use of CBCT images for the postoperative monitoring of root canal retreated teeth. However, only using postoperative PAs at follow-up will not provide accurate results due their low sensitivity, which is significantly less than that of CBCT scans. Monitoring of complete periapical osseous healing might be underestimated by conventional PAs, while CBCT might reveal the presence of periapical lesions [40, 41].

To maximise safety, the radiation dose used in for CBCT scans in the current study was small with a limited FOV [32]. The study also relied on the segmentation of CBCT images, which is a novel methodology to construct a 3D model in order to monitor periapical healing [42]. A previous study reported that the combination of human segmentation diagnostic systems was more sensitive for the detection and evaluation of healing of 153 periapical lesions using CBCT segmentation for lesion volume reduction [43].

The findings of the current trial indicate that REPs with either BC or PRF in the retreatment of mature permanent root-filled maxillary central incisor teeth with single straight canals in adolescents with periapical lesions was comparable in terms of clinical outcome and radiographic reduction of the width and size of periapical lesions after 12 months. Positive pulp testing responses to TPT and EPT occurred in significantly more teeth in the PRF technique. Consequently, the null hypotheses were partially rejected.

The results of the present study on root canal retreatment of mature teeth with REPs were consistent with those of primary root canal treatment over a 12-month follow-up period. For example, Arslan et al. [5] reported a clinical and radiographic success rate of 92.3% with an average age of 20.58 ± 2.53 years treated with REPs, compared to an 80% success of teeth treated conventionally. However, the apices of the treated teeth were violated with size 25 K-files. Youssef et al. [7] compared radiographic healing associated with two REP techniques (BC versus PRF) in 20 teeth (10 teeth per group) following the induction of intracanal bleeding with sizes 25 to 35 K-files. After 12 months, there was substantial healing of the periapical lesions with a reduction of PAI median scores from 3.5 and 4 to 2 and 1 in the BC and PRF groups, respectively. However, the scoring in these two studies was obtained from two-dimensional radiographs only.

Two articles have evaluated root canal retreatment with REPs for mature permanent teeth. One compared the efficacy of REPs using the BC technique to conventional root canal retreatment among early adolescents aged 11 to 17 years [27]. The clinical success rate of REPs was 93.9%, compared to 97% for root canal retreatment after a 12-month follow-up with a reduction in the median PAI scores from 3 to 1 mm after one year of follow-up [27]. These clinical findings are in line with the results of the present study; however, the assessment relied on the two-dimensional periapical images only. The second article was a case report of a 25-year-old female who was retreated with REPs and checked periodically for up to 36 months [44]. Clinical signs and symptoms subsided and traditional periapical and CBCT images revealed complete resolution of the periapical radiolucency and there was also a positive response to thermal and electric pulp testing.

The present study included a combination of thermal and electric pulp sensibility approaches, which has advantages [45] and widely used in previous investigations [46]. However, a positive response to pulp sensitivity tests after REPs as an indication of restoring pulp sensibility is controversial [47]. The positive responses to TPT and EPT might indicate that different stem cell lineages triggered from the periapical tissues, such as periodontal ligament SCs (PDLSCs) and bone marrow MSCs, have the capability of neuron differentiation [48]. Approximately half of the teeth treated with REPs responded positively to sensibility tests [49]. This was in line with the present findings, especially the BC results. The possible mechanisms by which regenerated cells respond can be attributed to the ability of SCAPs to release adenosine triphosphate (ATP) in response to cold triggers that activate the expression of neuron receptors. The other possible action might be associated with the direct effect of SCAPs on the activation and expression of thermoinductive ion channels in trigeminal neurons [49].

The current findings revealed more teeth had a pulp test response to EPT in PRF-retreated teeth compared to those in the BC group after 12 months of treatment. This was in agreement with previous reports that revealed positive pulp responses in 50% of PRF scaffolds compared to 20% for the BC technique [7] and 60% for the PRF technique [3]. The variation in released growth factors between the two REP techniques could explain the superiority of PRF in regaining pulp sensation over the BC group. PRF induces a variety of growth factors, including transforming growth factor-β (TGF-β), platelet-derived growth factor (PDGF), insulin growth factor 1, and basic fibroblast growth factor (bFGF), which mediate the neurogenesis process [50]. However, the main disadvantages of the PRF technique are that patients have to endure venepuncture to collect blood and the extended nature of the procedure. Histological analysis should be performed to identify the nature of regenerated/repaired tissue occupied the root canal space.

The current findings revealed that the preoperative diameter and volume of lesions had a significant effect on the reduction in their size following REPs. Lesion diameters below 3.85 mm demonstrated significantly greater reductions in size. This is consistent with the results of a previous trial of ten mature teeth with PALD scores ≥ 3 according to CBCT periapical index scores [51].

One of the challenges during REPs of mature teeth with long-standing periapical lesions is the induction of a significant amount of bleeding into the root canal space. This was found in two cases in the present trial. Eventually, the tip of the manual endodontic file was slightly bent after being dipped into 17% EDTA and an adequate amount of intracanal bleeding was achieved.

The main strengths of the current study are its novelty in using the segmentation of CBCT images with the aid of artificial intelligence for a more sensitive and accurate assessment of changes in the periapical lesion volume instead of relying solely on linear measures. This study may pave the way for a more comprehensive understanding of changes in periapical lesion size, especially in the new era of REPs for retreatment of mature teeth. Furthermore, the standardisation of the methodology to minimise the risk of potential confounders was also a strength. The main limitation of the study was the short follow-up period of 12 months and the lack of detail regarding the tissues formed in the root canal space. Ideally, histological analysis should be carried out to identify the nature of tissue formed in the root canal. Nevertheless, this study demonstrates the potential for expanding the use of different approaches of REPs in the retreatment of mature straight maxillary central incisor teeth with periapical lesions in adolescent patients. Further prospective randomized clinical trials with longer follow-ups and a wider age range are needed in an attempt to confirm the findings of the present study and their generalization to other settings.

## Conclusions

1. BC and PRF techniques of REPs are a viable option for retreating root filled mature straight permanent central incisor teeth in adolescents.
2. The BC and PRF techniques had comparable clinical and radiographic outcomes at 12 months.
3. Periapical lesion diameter and volume were significantly diminished after retreatment with BC or PRF scaffolds.
4. Teeth retreated with PRF had significantly more positive pulp test responses compared to teeth in the BC group.

## Data Availability

No datasets were generated or analysed during the current study.
